# Disinfection of lettuce using organic acids: an ecological analysis using 16S rRNA sequencing

**DOI:** 10.1039/c9ra03290h

**Published:** 2019-06-14

**Authors:** Jiayi Wang, Dongbing Tao, Shan Wang, Chen Li, Yanru Li, Fenge Zheng, Zhaoxia Wu

**Affiliations:** College of Food Science, Shenyang Agricultural University 120 Dongling Rd. Shenyang 110866 China wuzhaoxia@syau.edu.cn +86-130-6668-6988; Shenyang Product Quality Supervision and Inspection Institute Glide Rd Shenyang 110136 China

## Abstract

Organic acid disinfection efficacy has been previously estimated by analyzing microbial reduction on fresh produce. However, the effects of organic acids on the fresh produce microbiome are not considered for the evaluation of disinfection efficacy. Here, we studied the effects of seven generally recognized as safe organic acids (lactic, tartaric, acetic, propionic, malic, succinic, and citric acid), on the microbial counts and community on the surface of lettuce. The community was dominated by the following genera: *Xanthomonas* (24.73%), *Sphingomonas* (15.85%), *Massilia* (10.23%), *Alkanindiges* (9.00%), *Acinetobacter* (7.57%), and *Pseudomonas* (6.02%). Organic acid washing did not affect microbial diversity. Lactic acid was the most effective agent causing aerobic plate count reduction of 0.97 log CFU g^−1^; additionally, it increased the *Escherichia*–*Shigella* abundance from 0.77% to 3.29%. The relative abundance of *Xanthomonas*, a plant pathogen, was significantly increased by malic and propionic acid—propionic acid caused an increase from 24.73% to 47.53%. Microbial interaction analyses revealed the co-exclusion of *Xanthomonas* with the other core taxa, suggesting that the microbial distribution on the lettuce surface after disinfection carries a higher risk of quality loss. Therefore, the difference in disinfection efficacy of sanitizers was reflected in both microbial counts and bacterial community changes. We also propose a potential solution to control fresh produce safety and the rational use of sanitizers by collecting microbial diversity, composition, and count data from planting, transport, minimal processing, shelf and consumer storage, and gut digestion, and then using big data technology to develop a model to provide recommendations for sanitizer selection.

## Abstract 2

Comparing disinfection efficacies of same type sanitizer using a combined microbial reduction/16S rRNA analysis strategy.

## Graphical abstract

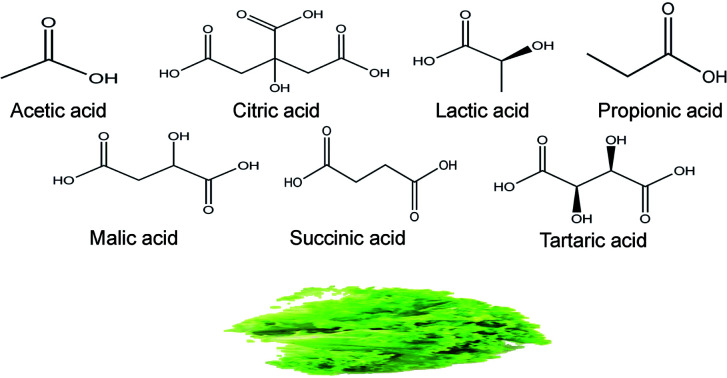

## Introduction

Consumption of fresh produce is an important part of the daily diet, providing necessary vitamins, minerals, and cellulose. The FDA recommends a daily intake of 3–5 different vegetables and 2–4 different fruits. Numerous disease outbreaks have been associated with foodborne pathogens (*e.g. Listeria monocytogenes*, *Escherichia coli*, and *Salmonella* spp.) that contaminate fresh produce. Therefore, disinfection is necessary before the produce is packaged for sale or before it is consumed. Although in recent years several novel disinfection technologies such as cold plasma, pulsed light, bacteriophages, and bioprotective microorganisms have been developed,^1^ chemical sanitizers such as chlorine, organic acid, ozone, hydrogen peroxide, sodium hypochlorite, and quaternary ammonium compounds are widely used owing to their high efficacy and low costs.^2^

Most organic acids elicit antibacterial activity by reducing environmental and cellular pH, and increasing anion accumulation and are listed as generally recognized as safe (GRAS) by the FDA.^3,4^ Acetic, lactic, and citric acids are commonly used in the minimal processing industry.^5^ Acetic and citric acids are suitable for use at the ready-to-eat stage and are frequently used in the form of vinegar and lemon juice. Other organic acids such as malic acid, propionic acid, succinic acid, and tartaric acid are also used as sanitizers and are listed as “direct food substances affirmed as GRAS” by the FDA.^2,6,7^

The dissociation constant (p*K*_a_) is a key indicator to evaluate the extent of dissociation of organic acids in aqueous solution. This value is dependent on the pH and is independent of acid concentration.^8^ The antibacterial activities of organic acids are traditionally attributed to cellular anion accumulation, which is determined by the proportion of undissociated molecules. Compared to the dissociated anions, undissociated acidic molecules have stronger lipophilicity, allowing them to penetrate the microbial cell membrane more easily.^8^ After penetration, the higher intracellular pH in the environment will promote acid molecule dissociation, and the dissociated anions will accumulate in the cell and exert toxic effects on DNA, RNA, and ATP synthesis.^9^ However, Ricke^10^ suggested that the relationship between energy dissipation and ATP production is complex and proposed that acid-sensitive protein denaturation and cell enlargement are major antibacterial mechanisms of action. Wang *et al.*^11^ also demonstrated the disruptive activity of lactic acid against the cytoplasmic membrane of the pathogens and the intracellular proteins. Comparison of the efficacies of different acids, however, must be made at the same concentration (mol L^−1^; *i.e.*, the p*K*_a_ value cannot be used to evaluate the antibacterial efficiency of organic acids under different molar concentrations).^9^ However, concentration is generally represented as w/w or v/v, which is followed in this study. In addition to p*K*_a_ values, differences in anion structure also affect the disinfection efficacy – for example, cinnamic acid (p*K*_a_ 4.4) is more effective than benzoic acid (p*K*_a_ 4.2).^8^ Moreover, not all organic acids exert their activity through anion accumulation – as an example, citric acid (CA) acts as a chelator to sequester metal ions (*e.g.*, Ca^2+^, Mg^2+^, Fe^3+^) required for bacterial homeostasis from the external medium.^9^

Therefore, the antibacterial activity of organic acids is complex and the differences in efficacy between different acids cannot be explained with a single mechanism. Hence, to evaluate the disinfection efficacy of organic acids, the traditional counts method should be considered along with the effects of organic acids on the ecology and environment of fresh produce. In recent years, the development of gene sequencing technology coupled with the powerful 16S rRNA analysis platform has enabled deep understanding of the bacterial communities on fresh produce. The objective of this study was to determine the effects of organic acids on lettuce surface microbiome using the 16S rRNA technology and combine it with the aerobic plate count results to evaluate the disinfection efficacies of different organic acids.

## Results and discussion

### Effects of organic acids on microbial counts and bacterial diversity of lettuce

**Fig. 1:**
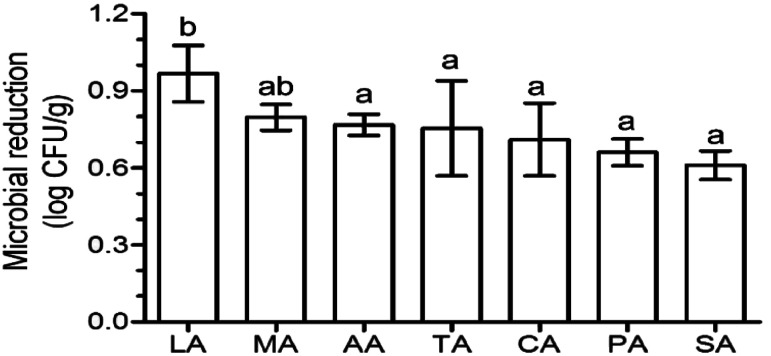
Effects of different organic acids on the naturally occurring microbes on the surface of lettuce. Different letters indicate significant differences (*p* < 0.05) based on Duncan's multiple range test. Columns represent mean values ± standard deviation. CA: citric acid, MA: malic acid, LA: lactic acid, TA: tartaric acid, SA: succinic acid, PA: propionic acid, AA: acetic acid.

Aerobic plate count is a key indicator of the extent of contamination in fresh produce, and the reduction in the aerobic plate count is generally used to evaluate the disinfection efficacy of sanitizers. After washing with organic acids, lactic acid (LA) induced the highest log reduction (0.97), followed by malic acid (MA) (0.80 log reduction) (Fig. 1). The log reduction of the other acids were significantly lower (*p* < 0.05) than that of LA, and were similar (*p* > 0.05) to each other – 0.77, 0.75, 0.71, 0.66, and 0.61 log reduction for acetic acid (AA), tartaric acid (TA), CA, (propionic acid) PA, and (succinic acid) SA, respectively (Fig. 1). This phenomenon was also found on *Escherichia coli* O157:H7 disinfection using organic acids, with a 1.5, 1.5, and 1.4 log reduction for CA, TA, and AA, respectively, which were significantly lower than that of LA (1.9 log reduction) and similar (*p* > 0.05) to that of MA (1.7 log reduction).^12^

**Fig. 2:**
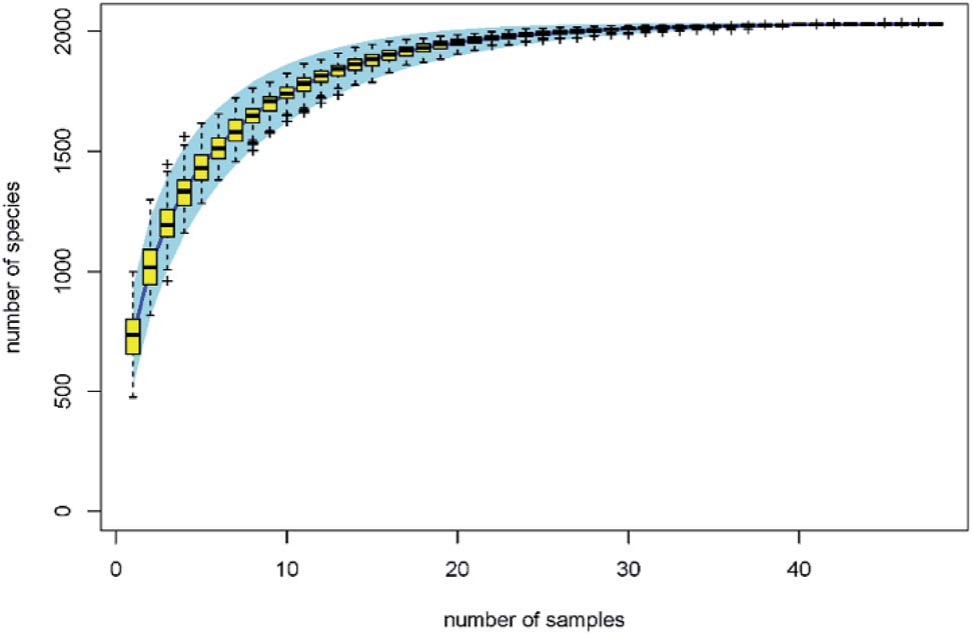
Specaccum species accumulation curves.

The traditional counting method is limited to understanding the microbial biodiversity on fresh produce. For example, the culture-dependent technology only quantifies 5% fungi belonging to specific taxonomic groups.^13^ Moreover, Lianou, *et al.*^8^ suggested that the disinfection efficacy of organic acids generally depends on the condition of the fresh produce ecology. With the development of gene sequencing technology, the microbial diversity on fresh produce has been determined in recent years. For example, the comprehensive investigation from Leff and Fierer^14^ indicates that the bacterial communities on each produce type (*i.e.* lettuce, apple, grapes, mushrooms, peach, pepper, spinach, strawberries, tomato, alfalfa sprouts, mung bean sprouts) were significantly distinct from one another. The effects of refrigeration on bacterial communities during the postharvest stage were also evaluated. The study from Lopez-Velasco *et al.*^15^ found that refrigeration can decrease the richness, diversity, and evenness of the microbial community in spinach, especially after storage for 15 days. Mycotoxin contamination risk during storage was also assessed by analyzing the fungal communities.^13^ However, as another key postharvest operation, we know far less about the disinfection effects on bacterial diversity of the produce. In this study, the effects of organic acids on bacterial diversity on the surface of lettuce were evaluated by calculating biodiversity estimators. The specaccum curve was steady as the sample numbers increased to 30, indicating that the 48 samples in this study are sufficient to evaluate the microbial diversity on lettuce surface (Fig. 2). The operational taxonomic unit (OTU) numbers of the treatments ranged from 625 to 811, and at the genus level, ranged from 592 to 765 (Table 1). Chao 1 and ACE estimator analysis showed that the values of these acids were not significantly different from that of the control group (Table 1), indicating that the organic acids can reduce the microbial counts without affecting the biodiversity.

**Table 1:** OTU numbers and biodiversity estimators of lettuce after treatment with an organic acid^a^

Treatment	Total OTU	Genus OTU	Chao 1	ACE
AA	731 ± 88	689 ± 73	851.39 ± 158.48^a^	881.46 ± 176.38^ab^
PA	625 ± 110	592 ± 101	731.77 ± 188.60^a^	739.22 ± 191.66^a^
LA	675 ± 90	638 ± 85	771.04 ± 163.33^a^	811.50 ± 190.27^ab^
TA	811 ± 136	765 ± 121	918.97 ± 160.87^a^	973.27 ± 173.99^b^
SA	758 ± 96	717 ± 91	880.87 ± 139.29^a^	914.67 ± 146.61^ab^
MA	738 ± 70	701 ± 58	840.10 ± 151.71^a^	863.30 ± 151.36^ab^
CA	758 ± 85	710 ± 78	868.05 ± 147.93^a^	902.00 ± 157.64^ab^
Control	749 ± 76	706 ± 71	832.02 ± 148.84^a^	858.62 ± 167.63^ab^

a Values (mean ± SD) in the same column with different letters are significantly different (*p* < 0.05). OTU: operational taxonomic unit; control: distilled water, CA: citric acid, MA: malic acid, LA: lactic acid, TA: tartaric acid, SA: succinic acid, PA: propionic acid, AA: acetic acid.

Most studies on the disinfection efficacy of sanitizers have reported that the natural microbial counts cannot be reduced by more than 3 log.^16^ This is mainly due to the irregular surface structure and stomata of the produce providing shelter to the bacteria.^5^ Reducing the microbial count to an attainable maximum limit and then employing the 16S rRNA technology to analyze the changes in biodiversity would reveal microbes that are resistant to disinfection.

### Effects of organic acids on bacterial composition

**Fig. 3:**
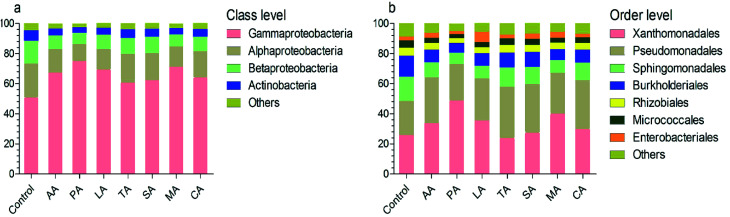
Bacterial composition after treatment with organic acids. (a) and (b) indicate the composition at the class and order level, respectively. Control: distilled water, CA: citric acid, MA: malic acid, LA: lactic acid, TA: tartaric acid, SA: succinic acid, PA: propionic acid, AA: acetic acid.

At the class level, the most numerous bacteria were Gammaproteobacteria, Alphaproteobacteria, Betaproteobacteria, and Actinobacteria (Fig. 3a). The relative abundance of the Alphaproteobacteria and Betaproteobacteria were significantly reduced after washing with organic acids (analyzed by LEfSe). However, the relative abundance of Gammaproteobacteria in lettuce washed with PA was significantly higher than that in other groups (*e.g.* 75.28% for PA *vs.* 51.14% for control). At the order level, the taxonomy was dominated by Xanthomonadales, Pseudomonadales, Sphingomonadales, Burkholderiales, Rhizobiales, Micrococcales, and Enterobacteriales (Fig. 3b). Among them, the composition of Sphingomonadales was significantly reduced by organic acids. Interestingly, the relative abundance of Enterobacteriales was only 2.63% in the control group; whereas previous studies have reported Enterobacteriales as the most abundantly represented order.^17^ This might be associated with the high abundance of Pseudomonadales (22.65%) and Burkholderiales (13.86%), which have been demonstrated to inhibit *Escherichia coli* O157:H7.^18^

Metastats was used to clarify the significant difference between each treatment at the genus level. In the control group, the core microbiome *Xanthomonas*, *Sphingomonas*, *Massilia*, *Alkanindiges*, *Acinetobacter*, and *Pseudomonas* accounted for 24.73%, 15.85%, 10.23%, 9.00%, 7.57%, and 6.02%, respectively (Table 2). Treatment with all organic acids other than TA significantly reduced the abundance of *Sphingomonas*. *Sphingomonas* has been reported to be enriched in the rhizosphere of lettuce independent of the soil type,^19^ and was found presenting on the surface of lettuce^13^ and tomato.^20^ It can metabolize various factors and stimulate plant growth,^20^ and has been shown to have plant-protective effects against *Pseudomonas syringae* through substrate competition.^21^ Similarly, *Massilia* is positively related to the root microbiome succession at an early stage during plant growth and has a potential to control plant pathogens such as *Pythium aphanidermatum*.^22^ Its abundance was also significantly reduced after washing with organic acids other than SA. It was found that the abundance of *Acinetobacter* and *Alkanindiges* was not decreased after washing. This might be advantageous since these two bacteria have been used to control fungal disease in plants and their abundance is considered as an indicator of good health in lettuce.^23,24^*Pseudomonas* is responsible for the loss of quality of fresh produce during the post-harvest stage. We found that its abundance was not decreased after treatment with organic acids.

**Table 2:** Relative abundance (genus level) of predominated taxa and *Escherichia*–*Shigella*^a^

Sanitizers	*Xanthomonas*	*Sphingomonas*	*Massilia*	*Alkanindiges*	*Acinetobacter*	*Pseudomonas*	*Escherichia*–*Shigella*
Control	24.73^a^	15.85^a^	10.23^a^	9.00^a^	7.57^ab^	6.02^ab^	0.77^a^
AA	32.50^ab^	9.78^bcd^	4.83^cd^	16.22^b^	8.64^b^	5.66^ab^	1.09^a^
PA	47.53^c^	7.09^d^	3.95^d^	13.80^b^	5.71^a^	4.87^ab^	0.58^a^
LA	34.48^abc^	7.99^bcd^	5.15^bcd^	14.92^ab^	7.46^ab^	5.46^ab^	3.29^a^
TA	22.86^ab^	12.42^ab^	5.88^bc^	16.38^b^	10.31^b^	7.41^ab^	0.81^a^
SA	26.00^ab^	11.15^bc^	6.93^ab^	16.10^b^	8.58^ab^	7.57^b^	1.36^a^
MA	38.44^bc^	8.17^cd^	4.48^cd^	14.72^b^	8.09^b^	4.32^a^	1.89^a^
CA	28.86^ab^	11.13^b^	5.47^bcd^	15.45^b^	12.61^b^	4.60^ab^	0.76^a^

a Significant difference was analyzed using Metastats. The values (mean) in the same column with different letters indicate significant differences (*p* < 0.05). Control: distilled water, CA: citric acid, MA: malic acid, LA: lactic acid, TA: tartaric acid, SA: succinic acid, PA: propionic acid, AA: acetic acid.

**Fig. 4:**
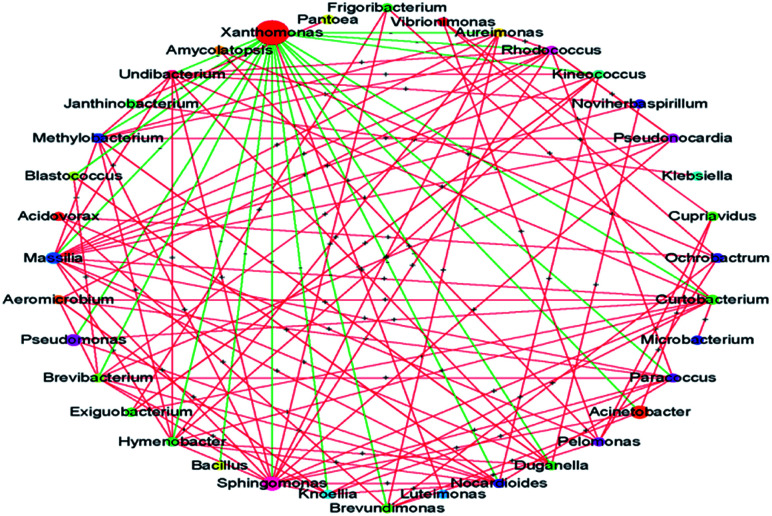
Microbial interaction at the genus level. The node size indicates the mean relative abundance of the taxa. The green and red lines represent co-exclusion and co-occurrence interaction, respectively.

A striking observation in this study was the increase in the abundance of *Xanthomonas*. Its abundance was significantly increased after treatment with PA and MA; in particular, after PA treatment, the abundance increased from 24.73% to 47.53%. Many *Xanthomonas* and *Pseudomonas* spp. initially cause water-soaked lesions followed by necrosis and chlorosis of the surface. Moreover, *Xanthomonas* was reported to induce hormonal imbalances in plants.^25^ The microbial interaction analysis showed the co-exclusion activity of *Xanthomonas* against other core microbiomes (Fig. 4), indicating that the abundance of *Xanthomonas* might be increased during subsequent transport, shelf, and consumer storage, decreasing the quality of the produce. In the study of Rastogi *et al.*,^26^ the presence of *Xanthomonas* was responsible for the leaf spot of lettuce and was positively correlated with the abundance of *Alkanindiges*. *Xanthomonas* can produce catalase and peroxidases to detoxify the reactive oxygen species (ROS) and other antimicrobial agents produced by plants. It is also a protective mechanism against host-produced photosensitizers and UV light.^25^ Thus, the increased abundance of *Xanthomonas* might be attributed to its strong resistance. The biodiversity and abundance of an environmental sample, such as lettuce in this study, is dependent on the origin, climate, season, transportation condition, and age.^26,27^ However, *Xanthomonas* was identified as the core microbe on lettuce across the world.^25^ Although the abundance of *Xanthomonas* was not significantly increased after treatment with the other five acids (*i.e.*, AA, TA, CA, LA, and SA), the effects of disinfection on *Xanthomonas* abundance should be carefully evaluated in future studies.

Furthermore, from a food processing perspective, browning is considered as major quality-loss in disinfected fresh produce. Based on our results, we hypothesize that browning caused by disinfection may resemble necrosis and chlorosis caused by *Xanthomonas*. In general, the relationship of pathogens with plant diseases is an important concern in farming practice. However, based on our results, we strongly recommend that researchers pay more attention to the relationship between sanitizers, plant pathogens, and produce quality.

No significant differences between microbial abundance in the AA, CA, and TA treatment groups were observed (Table 2). Interestingly, the microbial reduction for these three acids was also similar to each other (Fig. 1). It was also observed that *Escherichia*–*Shigella* accounted only for 0.7% abundance in the control group and was not significantly increased after disinfection. This might be a promising result; however, in many reports, the abundance of Enterobacteriales exceeded 20%. Furthermore, although LA induced the highest microbial reduction, it also caused the highest abundance of *Escherichia*–*Shigella* (3.29%). If the abundance of Enterobacteriales was similar to that in other studies, the fold change in *Escherichia*–*Shigella* abundance after LA treatment would be of serious concern. In the study from Poimenidou *et al.*,^28^*Escherichia coli* O157:H7 was undetected on lettuce washed with vinegar and stored; however, the counts in the sample treated with LA was approximately 3 log.

### Strategy for improving the disinfection of fresh produce

Although disinfection is a key operation during postharvest, it is non-selective, *i.e.*, it does not discriminate between background microbiota and pathogens and reduces the counts of all microbes. Some non-pathogenic microbes interact with and inhibit pathogenic microbes – for example, in tomato, the presence of *Enterobacter* and *Bacillus* spp. negatively affect the persistence of *Salmonella* spp.;^29^ the culturable *Pantoea ananatis* isolated from tomato leaves showed a protective value of 91.7 against gray mold.^20^ A previous study showed that the counts of *Listeria monocytogenes* on lettuce surface increased after disinfection with 0.5% AA, PA, and CA.^6^ This is mainly due to the unbalanced ecology environment after disinfection. Subsequently, during transport, shelf, and kitchen, fresh produce will more easily be colonized by pathogens. In addition to pathogen proliferation, it has been reported that irrespective of the initial aerobic plate counts, the plated counts of the sanitizer-treated group were similar to or higher than those of the control group during storage.^30–34^ Sanitizers are of two types – oxidizing agents and organic acids. Since they have different modes of action, a similar microbial reduction may be observed initially; while at the end of storage, significant differences in microbial counts are observed. For example, Poimenidou *et al.*^28^ found that the aerobic plate counts of lettuce washed with chlorinated water increased by 2.4 log after storage, whereas vinegar led to a 4 log reduction. This is due to the differences in the initial changes in bacterial composition and diversity induced by different sanitizers. However, in this study, we found that even sanitizers belonging the same type have a significantly different effect on the bacterial composition. Therefore, we recommended that changes in microbial composition and diversity, instead of a mere reduction in microbial counts should be considered when choosing sanitizers.

**Fig. 5:**
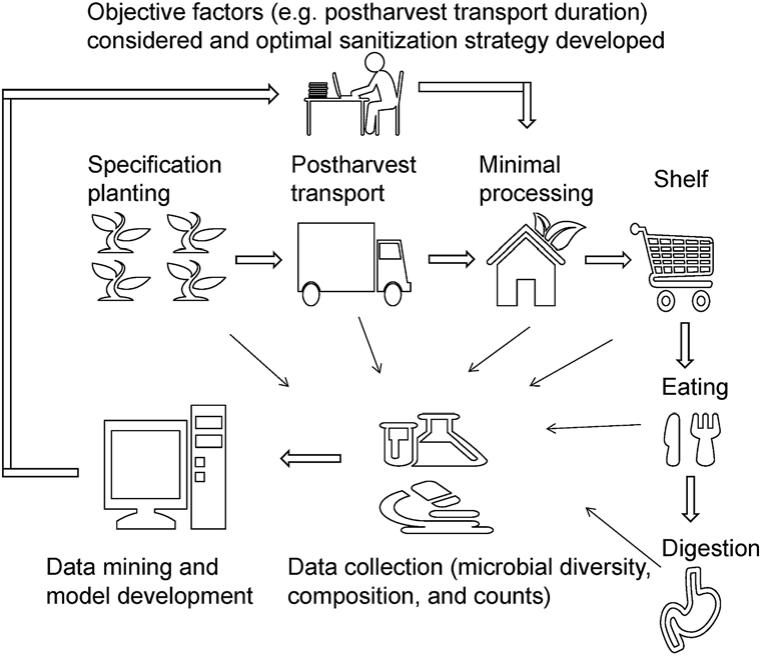
The proposed solution to improve fresh produce safety and sanitizer selection.

Microbial diversity and composition are dependent on many factors, and therefore choosing the best sanitizer may be challenging. In agriculture, centralized cultivation of specific crops is practiced in developed countries and is a trend in developing countries. The origin and cold chain for fresh produce (*e.g.* minimal processing industry) and the disinfection strategies are similar and well established. We propose an idea to improve the effect of disinfection on fresh produce, using big data technology integrating microbial diversity, composition, and counts, from planting to consumption (Fig. 5). Several pilot studies in recent years indicated that microbial diversity is responsible for the gut microbiome and immune response.^17,24,35^ Therefore, the changes in the gut microbiome should be considered and be integrated with farming practice and minimal processing.

## Experimental

### Sample preparation

Lettuce was purchased at a local market on the day of the experiment. The two outer leaves, inner baby leaves, and the stem were removed. The remaining leaves were then rinsed in tap water for one minute to remove the soil and then cut in pieces using a circle cutting edge (diameter of 5.2 cm).

Analytical grade AA, LA, CA, MA, PA, SA, and TA were purchased from Macklin (Shanghai, China). The p*K*_a_ values of TA, LA, CA, MA, SA, AA, and PA was 2.98, 3.08, 3.14, 3.46, 3.46, 4.21, 4.75, and 4.87, respectively. The pH of 1% TA, LA, CA, MA, SA, AA, and PA were 2.08, 2.15, 2.11, 2.22, 2.55, 2.66, and 2.78, respectively.

The cut lettuce samples were dipped in the acid solutions (18 ± 1 °C) at a ratio of 1 : 20 (w/v) and mechanically shaken for 1.5 min. Disinfected samples were washed with tap water for 15 s to remove the acid residue and drained until analysis. Six replicates were performed and samples washed with distilled water was used as the control.

### DNA extraction and aerobic count analysis

Each disinfected piece was divided into four even parts and 5 g leaf was taken in an Erlenmeyer flask containing 70 mL sterile 0.85% sodium chloride solution. The samples were shaken for 3 min at 260 rpm and were diluted 15-fold. Forty milliliters of the suspension was drawn with a sterile syringe and filtered through two 0.22 μm Millipore membranes (20 mL each). The membranes were used for total bacterial genomic DNA extraction using the Fast DNA SPIN extraction kits (MP Biomedicals, Santa Ana, CA, USA), following the manufacturer's instructions. The concentration of DNA was measured using a NanoDrop ND-1000 spectrophotometer (Thermo Fisher Scientific, Waltham, MA, USA) and agarose gel electrophoresis.

For counting analysis, 6 mL 15-fold diluted solution was added to 34 mL 0.85% sodium chloride solution to prepare a 100-fold diluted solution. Other serial dilutions were prepared as needed and uniformly mixed using a vortex shaker before use. One milliliter of the diluted solution was pour-plated onto an agar plate (Hopebio, Qingdao, China) for aerobic counts analysis, and the colonies were counted after a 2 day incubation period at 37 °C. Each replicate was analyzed in duplicate, and the results are expressed as log CFU g^−1^ reduction and the significant difference (*P* < 0.05) in means was analyzed by Duncan's multiple range tests, using SPSS v.20 software (SPSS Inc., Chicago, IL, USA). The aerobic plate count of the control group was 5.45 ± 0.12 log CFU g^−1^.

### Amplicon pyrosequencing

The V3–V4 region of the 16S rRNA genes in the extracted DNA was amplified by polymerase chain reaction (PCR) using 338F (5′-ACTCCTACGGGAGGCAGCA-3′) and 806R (5′-GGACTACHVGGGTWTCTAAT-3′) as the forward primer and reverse primer, respectively. Seven-bp barcodes were incorporated into the primers for multiplex sequencing. The PCR mixture contained 1 μL forward primer (10 μM), 1 μL reverse primer (10 μM), 2 μL DNA Template, 0.25 μL Q5 High-Fidelity DNA Polymerase (5 U μL^−1^), 5 μL Q5 reaction buffer (5×), 5 μL Q5 High-Fidelity GC buffer (5×), 2 μL dNTPs (2.5 mM), and 8.75 μL ddH2; the PCR conditions were as follows: initial denaturation at 98 °C for 2 min followed by 25 cycles of denaturation at 98 °C for 15 s, annealing at 55 °C for 30 s, and extension at 72 °C for 30 s, and a final extension at 72 °C for 5 min. The resulting PCR amplicons were purified using the Agencourt AMPure Beads (Beckman Coulter, Indianapolis, IN) and quantified using the PicoGreen dsDNA Assay Kit (Invitrogen, Carlsbad, CA, USA). After pooling in equal amounts, the obtained amplicons were sequenced using the Illumina MiSeq platform at Shanghai Personal Biotechnology Co., Ltd (Shanghai, China).

### Sequence analyses

The QIIME v. 1.8.0 pipeline was employed to control the quality of the sequencing data, and the process has been described by Caporaso *et al.*^36^ In brief, the data with exact matches were classified as high-quality sequences, and the data with a length shorter than 150 bp, average Phred scores less than 20, and containing ambiguous bases and mononucleotide repeats (>8 bp) was classified as low-quality sequences.^37^ After FLASH^38^ pair-end reads and chimera detection, the obtained sequences were clustered as OTUs at 97% sequence identity using UCLUST.^39^ Then, default parameters were used to select a representative sequence, which was imported to the BLAST system to classify the OTU against the Greengenes Database using the best hit. The resulting OTUs containing less than 0.0001% of the total sequences (all samples) were discharged. The sequencing depth difference across the samples was minimized by resampling 100 OTU subsets under 90% sequencing depth and then averaging them to generate a rounded rarefied table for further analysis. All raw sequencing data have been deposited into the NCBI Sequence Read Archive (SRA) database with accession number SRP199012.

### Bioinformatic analyses

The OTU table was used for bioinformatic analyses, and the analyses were mainly performed using QIIME, Mothur, and R packages (v3.2.0). The Specaccum species accumulation curves were plotted using the R software. The alpha diversity index (*i.e.*, Chao 1 and ACE estimator) was calculated using QIIME. The relative abundance and compositions were visualized using the R software. The significant difference between each group was analyzed by LEfSe using the default parameters,^40^ and at the genus level, was analyzed using Metastats.^41^ The co-occurrence or co-exclusion interaction of the dominant (relative abundance top 50) microbe was analyzed based on the relative Spearman's rank (|rho| > 0.6; *P* < 0.01) that was calculated using Mothur software. The interaction was visualized using Cytoscape.

## Conclusions

In this study, microbial counts and diversity were detected simultaneously. The results showed that the difference in disinfection efficacy of organic acids was reflected in not only microbial counts but also changes in the bacterial community. However, because of limitations of the counting method, we were unable to correlate the counting data with the relative abundance, which would elucidate the resistance and sensitivity of each microbial taxon to a single sanitizer. Then, sanitizer formulations can be redesigned to improve disinfection efficacy. We also found that PA and MA increased the abundance of *Xanthomonas*, and this increase may show co-exclusion activity against other microbes and cause quality loss. This speculation has not been confirmed by storage experiments. Therefore, these results only illustrate the potential risk of quality loss after disinfection. Nevertheless, when the disinfection efficiency is similar, we can choose sanitizers that are cheaper (compared with PA and MA) and do not pose a quality-loss risk, such as CA. In subsequent studies, we plan to conduct a storage experiment, sample the brown spots and compare their microbial diversity with that of healthy leaf tissue, and explore the relationships among sanitizers, micro-ecological changes, and quality loss.

## Conflicts of interest

There are no conflicts to declare.

## Supplementary Material
